# Draft Genome Sequence of a Multiple Antibiotic Resistant Staphylococcus aureus NCTC 6571-UB Laboratory Strain

**DOI:** 10.1128/mra.00448-22

**Published:** 2022-08-23

**Authors:** Paul Norton, Kah-Ooi Chua, Maria Katsikogianni, M. Julie Thornton, Kok-Gan Chan, Chien-Yi Chang

**Affiliations:** a Centre for Skin Sciences, Faculty of Life Sciences, University of Bradford, Bradford, United Kingdom; b Centre for Research in Biotechnology for Agriculture, University of Malaya, Kuala Lumpur, Malaysia; c Polymer and Biomaterials Research Centre, School of Chemistry and Bioscience, University of Bradford, Bradford, United Kingdom; d Institute of Biological Sciences, Faculty of Science, University of Malaya, Kuala Lumpur, Malaysia; e International Genome Centre, Jiangsu University, Zhenjiang, China; f School of Dental Sciences, Newcastle University, Newcastle upon Tyne, United Kingdom; University of Rochester School of Medicine and Dentistry

## Abstract

We report the draft genome sequence of the laboratory strain Staphylococcus aureus NCTC 6571-UB, a strain that was derived from S. aureus NCTC 6571. This strain was selected for sequencing in order to provide information on the genome dynamics and the acquired resistance genes for penicillin G, trimethoprim, and sulfamethoxazole resistance.

## ANNOUNCEMENT

Staphylococcus aureus NCTC 6571 Oxford strain is a reference strain for penicillin sensitivity bioassays that was first deposited in the National Collection of Type Cultures (NCTC) by N. G. Heatley in 1943 (1). S. aureus NCTC 6571-UB, a strain in our collection that originated from NCTC 6571, was assessed for its antibiotic susceptibility via the standard Kirby-Bauer disc diffusion assay (2) on Oxoid tryptone soy (TS) agar at 37°C for 24 h. The strain was found to be resistant to penicillin G, trimethoprim, and sulfamethoxazole, indicating that it has multiple antibiotic resistance phenotypes (Table 1). Therefore, the genome of NCTC 6571-UB was sequenced for a comparative analysis with the published genome of NCTC 6571 (GenBank accession number GCA_900457695).

**TABLE 1:** S. aureus NCTC 6571-UB antibiotic resistance profile

Antibiotic	Amt in disc^*a*^	Bacterial susceptibility^*b*^	Zone of inhibition (mm)^*c*^	Resistant colonies^*d*^
Chloramphenicol	25 μg	Intermediate	16	N
Clindamycin	2 μg	Susceptible	22	N
Erythromycin	5 μg	Intermediate	20	N
Fusidic acid	10 μg	Susceptible	22	N
Novobiocin	5 μg	Susceptible	16	N
Oxacillin	5 μg	Susceptible	13	Y
Penicillin G	1 unit	Resistant	14	Y
Streptomycin	10 μg	Intermediate	14	N
Sulfamethoxazole	25 μg	Resistant	NA	N
Tetracycline	10 μg	Susceptible	20	N
Tetracycline	25 μg	Susceptible	30	N
Trimethoprim	25 μg	Resistant	NA	N

a The filter paper discs are about 6 mm in diameter.

b Resistant, intermediate, and susceptible are degrees of resistance to the corresponding antimicrobials according to Clinical and Laboratory Standards Institute guidelines (2).

c NA, not applicable (no zone of inhibition).

d Y, yes; N, no.

The genomic DNA (gDNA) of NCTC 6571-UB was extracted from a 37°C overnight TS broth culture. Cells were lysed in Tris buffer (10 mM Tris-HCl pH 8.0) containing lysozyme. RNase A (0.1 mg/mL), proteinase K (0.1 mg/mL), and SDS (0.5% [vol/vol]) were subsequently added. gDNA was purified using SPRI beads (Beckman Coulter, USA) and sequenced by MicrobesNG (Birmingham, UK). Multiple gDNA libraries were prepared using the Nextera XT library preparation kit (Illumina, San Diego, CA, USA), quantified using the Kapa Biosystems library quantification kit for Illumina, pooled, and sequenced with an Illumina NovaSeq sequencer (250-bp paired-end read setting). The raw data were quality filtered using Trimmomatic v0.36 (3) and *de novo* assembled using SPAdes v3.7 (4). The assembled genome was assessed for quality using QUAST v5.2.2 (5) and for completeness using BUSCO v5.3.2 (6). Genome annotation was performed by the Prokaryotic Genome Annotation Pipeline (PGAP) (7), Rapid Annotations using Subsystems Technology (RAST) (8, 9), and Prokka v1.14.6 (10). Default parameters were used for all software.

The sequencing resulted in 1,805,525 raw reads. The assembled draft genome of NCTC 6571-UB had a total length of 2,809,965 bp, with a G+C content of 32.7%, and it consisted of 65 contigs (*N*_50_, 125,553 bp; *N*_75_, 77,862 bp), with a total of 2,661 coding DNA sequences, 58 tRNA genes, and 9 rRNA genes. The average coverage of the draft genome was 30×, and BUSCO analysis revealed 99.8% completeness. NCTC 6571-UB shared 99.86% 16S rRNA gene sequence similarity with the type strain S. aureus subsp. *aureus* DSM 20231 GenBank accession number AMYL01000007, but their genomes exhibited 97.5% average nucleotide identity (ANI) (11) and 76.8% *in silico* DNA-DNA hybridization (DDH) (accessed at https://ggdc.dsmz.de/ggdc.php#) (12). These findings indicate that NCTC 6571-UB is an S. aureus species. The strain also shared 100% 16S rRNA gene sequence similarity, 100% ANI, and 100% *in silico* DDH values with the NCTC 6571 Oxford strain (GenBank accession number GCA_900457695.1).

NCTC 6571-UB harbored a prophage-associated metallo-β-lactamase superfamily domain protein (locus tag M3M53_RS02030), which could confer penicillin resistance (13). Compared to the Oxford strain, NCTC 6571-UB did not show mutations in the genes for dihydrofolate reductase (locus tag M3M53_RS08060) and dihydropteroate synthase (locus tag M3M53_RS12540), which are responsible for staphylococcal resistance to trimethoprim and sulfamethoxazole, respectively (14, 15). Our analysis also revealed no mutation in the thymidylate synthase gene (locus tag M3M53_RS08065) (16) and no plasmid-borne dihydrofolate reductase (17) that could contribute to trimethoprim-sulfamethoxazole resistance. Overall, the resistance phenotypes of NCTC 6571-UB may be mediated by other mechanisms, and further study is required to confirm this hypothesis.

### Data availability.

The whole-genome sequencing project was deposited in GenBank under the BioProject accession number PRJNA835436, with BioSample and Sequence Read Archive (SRA) accession numbers SAMN28102073 and SRR19138527, respectively. The whole-genome sequence is available in GenBank under the accession number JAMFMC000000000.1.
